# Endocrine therapy-resistant breast cancer model cells are inhibited by soybean glyceollin I through *Eleanor* non-coding RNA

**DOI:** 10.1038/s41598-018-33227-y

**Published:** 2018-10-12

**Authors:** Tatsuro Yamamoto, Chiyomi Sakamoto, Hiroaki Tachiwana, Mitsuru Kumabe, Toshiro Matsui, Tadatoshi Yamashita, Masatoshi Shinagawa, Koji Ochiai, Noriko Saitoh, Mitsuyoshi Nakao

**Affiliations:** 1 Department of Medical Cell Biology, Institute of Molecular Embryology and Genetics, Kumamoto University, 2-2-1 Honjo, Chuo-ku, Kumamoto, 860-0811 Japan; 2 Division of Cancer Biology, The Cancer Institute of JFCR, 3-8-31 Ariake, Koto-ku, Tokyo, 135–8550 Japan; 3 Faculty of Agriculture, Graduate School of Kyushu University, 744 Mototoka, Nishi-ku, Fukuoka, 819-0395 Japan; 4 Tokiwa Phytochemical Co. Ltd., 158 Kinoko, Sakura-shi, Chiba, 285–0801 Japan; 5 Kajitsudo Co., Ltd, 1155-5, Tabaru, Mashiki-machi, Kamimashiki-gun, Kumamoto, 861–2202 Japan; 6 Department of Oral and Maxillofacial Surgery, Faculty of Life Sciences, Kumamoto University, Kumamoto, Japan

## Abstract

Long-term estrogen deprivation (LTED) of an estrogen receptor (ER) α-positive breast cancer cell line recapitulates cancer cells that have acquired estrogen-independent cell proliferation and endocrine therapy resistance. Previously, we have shown that a cluster of non-coding RNAs, *Eleanors* (*ESR1 locus enhancing and activating non-coding RNAs*) formed RNA cloud and upregulated the *ESR1* gene in the nuclei of LTED cells. *Eleanors* were inhibited by resveratrol through ER. Here we prepared another polyphenol, glyceollin I from stressed soybeans, and identified it as a major inhibitor of the *Eleanor* RNA cloud and *ESR1* mRNA transcription. The inhibition was independent of ER, unlike one by resveratrol. This was consistent with a distinct tertiary structure of glyceollin I for ER binding. Glyceollin I preferentially inhibited the growth of LTED cells and induced apoptosis. Our results suggest that glyceollin I has a novel role in LTED cell inhibition through *Eleanors*. In other words, LTED cells or endocrine therapy-resistant breast cancer cells may be ready for apoptosis, which can be triggered with polyphenols both in ER-dependent and ER-independent manners.

## Introduction

Breast cancers expressing estrogen receptor-α (ER) depend on estrogen for cellular growth and survival. Endocrine therapies, such as those using aromatase inhibitor (AI) to block estrogen production, are effective for treating ER-positive breast cancers^1^. However, these treatments are often followed by disease recurrence, because most tumors acquire the ability to proliferate under estrogen depletion. During this process, upregulation of ER occurs, which may make the cells hypersensitive to residual amounts of estrogen and allowing them to grow^1–3^.

MCF7, a human breast cancer cell line, is ER positive and acquires estrogen-independent proliferation when it is cultured under an estrogen-deprived condition for a prolonged period (long-term estrogen deprivation; LTED)^4,5^. LTED adaptation is a well-established cellular model that recapitulates acquisition of AI resistance or postmenopausal tumorigenesis^4–9^. Previous studies have shown that the *ESR1* gene encoding ER was activated during LTED adaptation^7^, which was critical for LTED survival^10^. The upregulation of *ESR1* was induced and maintained by transcription of clusters of non-coding (ncRNAs) called *Eleanors* (*ESR1 locus enhancing and activating non-coding RNAs*) from a large chromatin domain including the *ESR1* gene. Fluorescence *in situ* hybridization (FISH) analysis showed that *Eleanors* were localized at the site of their own transcription, resulting in the formation of distinct RNA foci in the nucleus, called the *Eleanor* RNA cloud.

We have also shown that resveratrol, a polyphenol, dramatically suppresses the *Eleanor* RNA cloud through its estrogenic effect^10^. Resveratrol is structurally related to estrogen^11^, which induces apoptosis in LTED cells^12,13^. These results may reflect well-known estrogen additive therapy, in which high doses of estrogen can promote tumor regression in postmenopausal women with recurrent ER-positive breast cancer who had previously received endocrine therapies^14–18^. The treatment is paradoxical, because estrogen generally enhances tumor cell growth and prevents apoptosis. It is anticipated that the analysis of estrogen and its related compounds will elucidate the mechanism for the additive therapy and cancer recurrence and identify new therapeutic targets.

Phytoalexins are small natural compounds that are synthesized *de novo* as a self-defense system in plants after experiencing stresses, including infection, wounding, freezing, UV light, and microbial infection^19,20^. The inducible soybean phytoalexins also have multifunctional health-promoting properties as regulators of inflammatory responses, glucose metabolism, antimicrobials, antioxidants, and other processes^21,22^. One representative group of phytoalexins, the glyceollins, is structurally related to estrogen. Glyceollin I has been shown to exert an anti-estrogenic effect by competing with endogenous estrogen and suppressing breast and ovarian tumorigenesis^19,23,24^. Besides, alternate mechanisms have been suggested, in which glyceollin I targets estrogen-independent pathways to inhibit the proliferation of breast cancer cells^25–29^. Currently, it is largely unknown whether glyceollin I has a biological effect on LTED cells, as resveratrol and estrogen do.

Here we prepared a mixture of glyceollins from activated soybeans and identified glyceollin I as a suppressor of LTED cells. Glyceollins regressed *Eleanor* RNA cloud formation, *ESR1* mRNA transcription, and cell proliferation. Notably, glyceollin I preferentially inhibited the cell growth of LTED cells compared with MCF7 and normal fibroblast IMR-90 cells. Glyceollin I and resveratrol induced LTED cell death. Glyceollin I was unique in that it suppressed LTED cells independently of ER. Overall, our data suggest that LTED cells are fragile and their cell death can be triggered with polyphenols through repressing *Eleanor* RNA.

## Results

### Detection of *Eleanor*-targeted inhibitory activity in activated soybeans

**Figure 1:**
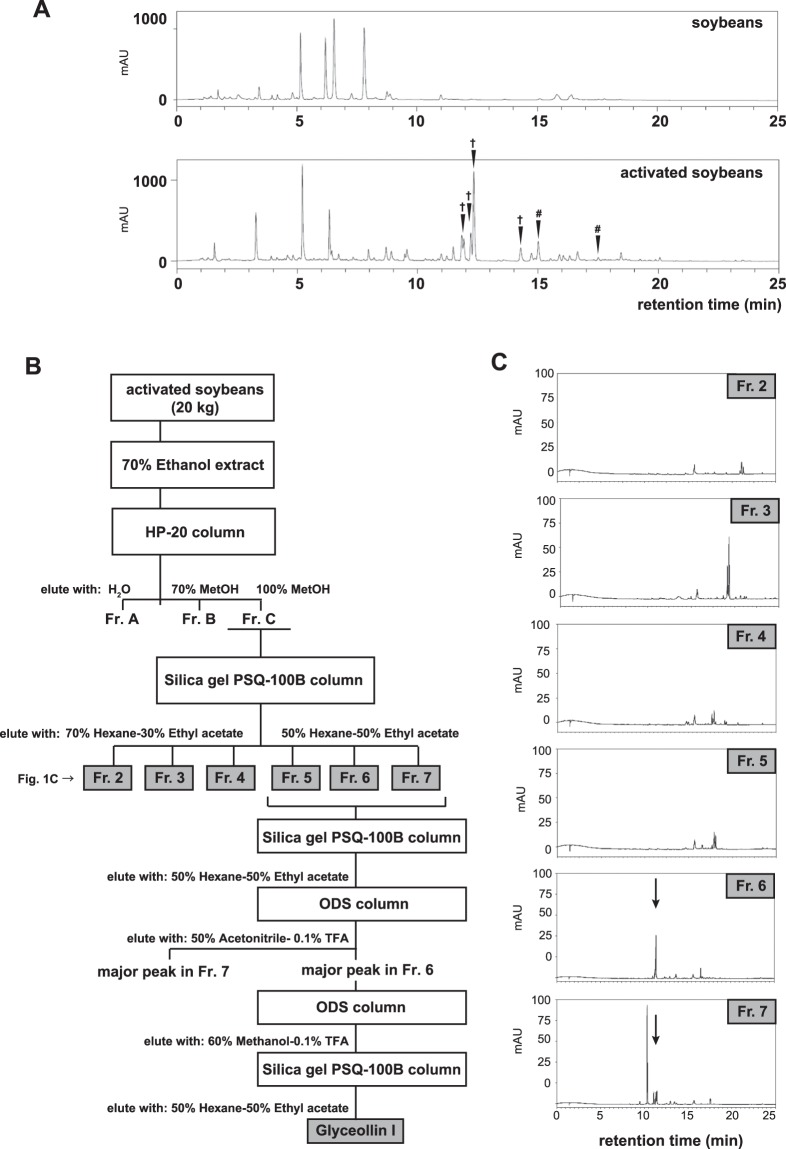
Fractionation of lysate derived from the activated soybean. (**A**) HPLC chromatogram of soybean extracts. As indicated by the arrows, phytoalexins were induced in the soybean seeds that were activated with *Aspergillus oryzae*, and corresponding peaks appeared at 11 to 13 min retention time. Water and acetonitrile acidified with acetic acid were used as solvents to apply a linear gradient over time (see Materials and Methods for detail). Components in peaks marked with †and # were estimated to be glyceollins and prenylated isoflavones, respectively. AU; mAU milli-Absorbance Units. (**B**) Scheme for phytoalexin preparation. (**C**) Acquity UPLC chromatogram of the indicated fractions, which were analyzed for their cancer repression assays in Fig. 2. The arrows indicate a common phytoalexin component in Fr. 6 and Fr. 7.

It has been shown that various natural compounds derived from soybeans exert anti-estrogenic effects on ER-positive breast cancer cells^19,23,24^, which prompted us to investigate their effects specific to an endocrine therapy-resistant breast cancer cell model, LTED cells. For this purpose, we analyzed phytoalexins produced in soybeans that were activated at their germination with a combination of stresses; no light, higher temperature (27 °C), and fungi inoculation (*Aspergillus oryzae*). The extract was prepared from the soybeans with 70% ethanol and chromatographed over a high-performance liquid chromatography (HPLC) with the Acquity UPLC BEH shield RP18. The HPLC profile showed extra peaks in the activated soybeans at 11 to 13 min retention time, which correspond to phytoalexins including glyceollins and prenylated isoflavones (arrows in Fig. 1A)^30,31^. To analyze the effects of these compounds on the breast cancer cell lines, we prepared a large amount of phytoalexins from 20 kg soybeans, separated in fraction (Fr.) 2 through 7 with biochemical columns with Styrene body (HP-20) and silica gel (PSQ-100B) synthetic adsorbents (Fig. 1B). A portion of each fraction was profiled with analytical HPLC with the Acquity UPLC BEH shield RP18 (Fig. 1C**)**. The phytoalexins eluted at 11–13 min retention time with this column (Fig. 1A) were mainly collected in Frs. 6 and 7 (arrows in Fig. 1C).

**Figure 2:**
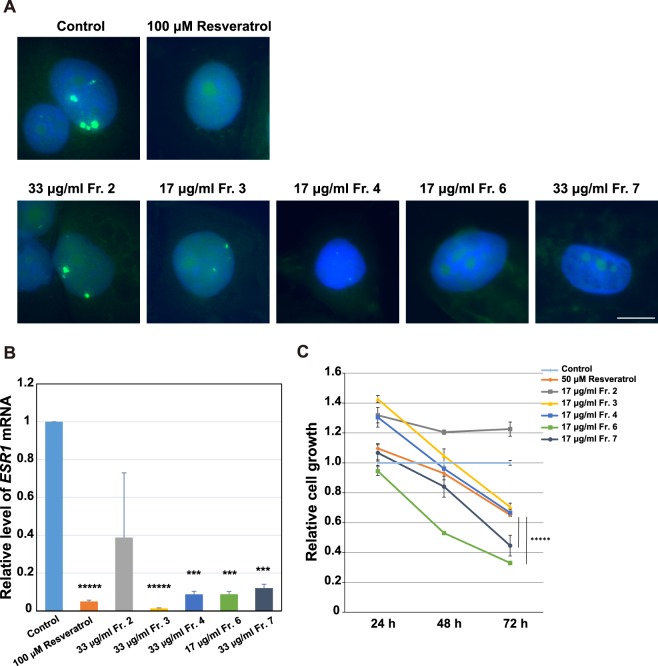
Inhibition of the *Eleanor* RNA cloud, *ESR1* mRNA, and LTED cell proliferation by the phytoalexin fractions. (**A**) The *Eleanor* RNA cloud regressed upon treatment with the biochemical fractions of the soybean extracts. LTED cells were treated with each phytoalexin fraction (Fig. 1) and subjected to RNA FISH to visualize *Eleanor* foci (green). The nucleus was counterstained with DAPI (blue). Resveratrol has been previously shown to inhibit *Eleanors*^10^. Scale bar, 10 µm. (**B**) Transcription of *ESR1* mRNA was inhibited with the extract fractions. Quantitative RT-PCR was performed to measure relative *ESR1* mRNA levels in LTED cells treated as indicated. Values were normalized against *GAPDH* mRNA, and values for cells treated with DMSO (control) were set to 1. The bars represent the means ± S.D. *n* > 3. Resveratrol has been previously shown to efficiently inhibit *ESR1* mRNA^10^. ***p < 0.001; *****p < 0.00001. (**C**) LTED cell proliferation was inhibited by the extract fractions. LTED cells were treated as indicated, and the viabilities were measured. Resveratrol has been previously shown to inhibit LTED cell proliferation^10^ *****p < 0.00001.

LTED cells represent breast cancer relapsed from heavy exposure to endocrine therapy and are established from the human ER-positive breast cancer cell line MCF7 by culturing without estrogen for a long period, typically 3–4 months. To address how the partially purified phytoalexins act on LTED cells, we analyzed the following three aspects. One was the inhibitory property on the *Eleanor* RNA cloud, which is composed of a cluster of non-coding RNAs that emerged from a 0.7 Mb chromatin domain containing genes upregulated in LTED cells^10,32^. We performed RNA FISH to visualize a portion of the *Eleanors*, including introns 1–5 of the *ESR1* pre-mRNA, as well as *u-Eleanor*, which is transcribed from chr 6q25 and is at least 1000 nt in length, and functions as an enhancer RNA in LTED cells^10^. Our RNA FISH showed typical enlarged RNA cloud (green) in control LTED cell nuclei (blue) that were treated for 24 h with DMSO, a solvent for each phytoalexin fraction (Fig. 2A). Treatment of the LTED cells with soybean-derived Frs. 3, 4, 6, and 7, but not Fr. 2, resulted in marked regression of the foci, similarly to the treatment with estrogen (Fig. S1A) and resveratrol, known as an *Eleanor* inhibitor^10^ (Fig. 2A).

The second criteria were an inhibitory effect on *ESR1* mRNA level. Previously, we have shown that upregulation of *ESR1* mRNA was essential for LTED cell proliferation, supported by *Eleanors* and inhibited with resveratrol^10^. Consistently with loss of the *Eleanor* RNA cloud in Fig. 2A, *ESR1* mRNA level decreased by treatment with Frs. 3, 4, 6, and 7 for 24 h, to a comparable degree with the ones with estrogen (Supplementary Fig. S1B) and resveratrol treatments (Fig. 2B).

The last criteria were an effect on LTED cell proliferation (Fig. 2C). First, we confirmed that the growth of LTED cells was effectively inhibited by resveratrol and estrogen (Fig. 2C, orange bar and Supplementary Fig. S1C,D), as previously shown^10,12^. Then we tested the soybean extract fractions, and found that all but Fr. 2 efficiently inhibited cell growth in a time-dependent manner. The effects of Frs. 6 and 7 were more than resveratrol that was previously shown to inhibit LTED cell growth^10^. Altogether, we concluded that Fr. 6 was the most potent, regarding LTED cell inhibition through suppression of *Eleanors* and *ESR1* mRNA.

### Structural determination of the *Eleanor* inhibitor as glyceollin I by NMR and TOF-MS

**Figure 3:**
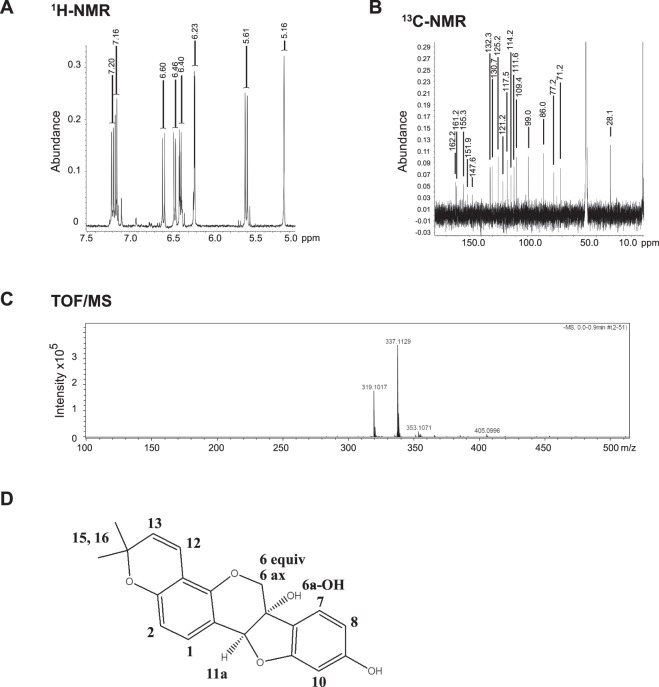
Structural determination of Fr. 6 as glyceollin I with NMR and TOF-MS. One-dimensional ^1^H-NMR (**A**) and ^13^C-NMR (**B**) measurements of Fr. 6 were performed in methanol-*d*_4_. **(C)** Monoisotopic molecular ion of 337.1129 *m/z* corresponding to C_20_H_17_O_5_^−^ as [M-H]^−^ (theoretical *m/z*: 337.1070) by TOF-MS analysis. (**D**) Chemical structure of glyceollin I.

To identify precisely which phytoalexin has potency to repress LTED cells, we identified the most major compound in Fr. 6 by NMR and time-of-flight–mass spectrometry (TOF-MS) analyses. 1D-^1^H-NMR spectra showed characteristic doublets of aromatic ring protons in the range of 5.64–7.20 ppm and confirmed the complete agreement of chemical shifts with the reported glyceollin I in the solvent system methanol-*d*_4_^33^ **(**Fig. 3A**)**. In addition, chemical shifts of the carbon atom in 1D-^13^C-NMR spectra were in good agreement with previous reports^33^ **(**Fig. 3B**)**, as following. Glyceollin I: ^1^H-NMR (methanol-*d*_4_, 400 MHz in ppm) 7.20 (H-1, *d*, *J* = 8.2 Hz), 7.16 (H-7, *d*, *J* = 8.2 Hz), 6.60 (H-12, *d*, *J* = 10.1 Hz), 6.46 (H-2, *d*, *J* = 8.2 Hz), 6.40 (H-8, *m*), 6.23 (H-10, *d*, *J* = 2.3 Hz), 5.61 (H-13, *d*, *J* = 10.1 Hz), 5.16 (H-6a, 11a, *s*), 4.16 (H-6 equiv, *dd*, *J* = 0.9, 11.5 Hz), 3.93 (H-6 ax, *d*, *J* = 11.5 Hz), 1.36 and 1.38 (H16/15, s); ^13^C-NMR (methanol-*d*_4_, 100 MHz in ppm) 162.2, 161.2, 155.3, 151.9, 147.6, 132.3, 130.7, 125.2, 121.2, 117.5, 114.2, 111.6, 109.4, 99.0, 86.0, 77.2, 71.2, 28.1. Further, a molecular ion peak of 337.1129 *m/z* in negative TOF-MS corresponded to C_20_H_17_O_5_^−^ as [M-H]^−^ (theoretical *m/z*: 337.1070) (Fig. 3C). Therefore, the compound was assigned to glyceollin I (molecular weight: 338.3539, C_20_H_18_O_5_) (Fig. 3D).

### Glyceollin I preferentially inhibits proliferation of LTED cells

**Figure 4:**
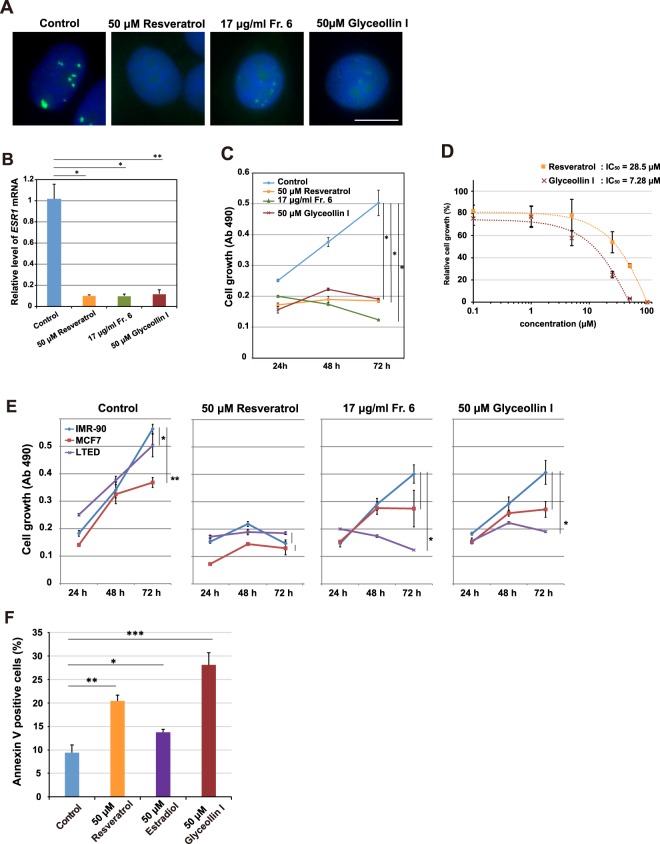
Purified glyceollin I preferentially inhibits LTED cells and induces apoptosis. (**A**) The *Eleanor* RNA cloud was inhibited with glyceollin I purified from activated soybeans, equivalently to a known inhibitor, resveratrol^10^. LTED cells were treated as indicated and subjected to RNA FISH to visualize *Eleanor* foci (green). The nucleus was counterstained with DAPI (blue). Scale bar, 10 µm. (**B**) Transcription of *ESR1* mRNA was inhibited with glyceollin I. Quantitative RT-PCR was performed to measure relative *ESR1* mRNA levels in LTED cells treated as indicated. Values were normalized against *GAPDH* mRNA, and values for cells treated with DMSO (control) were set to 1. The bars represent the means ± S.D. *n* >3, *p < 0.05; **p < 0.01. (**C**) LTED cell proliferation was inhibited by glyceollin I. LTED cells were treated as indicated, and cell viabilities were measured. The values represent the means ± S.D. *n* >3, *p < 0.05. (**D**) LTED cell growth was inhibited by glyceollin I in a dose-dependent manner. The inhibition by glyceollin I was more effective than that by resveratrol. The value of DMSO treatment (control) was set to 100. The values represent the means ± S.D. *n* = 3. (**E**) Glyceollin I preferentially inhibited growth of LTED cells. IMR-90 (normal human fibroblast derived from fetal lung), MCF7 and LTED cells were treated as indicated, and cell viabilities were measured with a colorimetric assay at Ab 490 nm (Kit-8). *p < 0.05; **p < 0.01. (**F**) Apoptosis was induced in LTED cells by glyceollin I. Cells were treated with indicated compounds for 24 h, stained with Annexin V. FACS analysis showed that glyceollin I and resveratrol induced apoptosis, as estradiol. The values represent the means ± S.D. *n* = 3, *p < 0.05; **p < 0.01, ***p < 0.001. Corresponding FACS data are shown in Supplementary Fig. S4.

Using the purified glyceollin I, we investigated its effect on the *Eleanor* RNA cloud, *ESR1* mRNA and cell proliferation (Fig. 4). Our RNA FISH analysis showed that glyceollin I inhibited RNA cloud formation as efficiently as resveratrol with 24 h treatment at a 50 μM concentration (Fig. 4A). Glyceollin I also significantly reduced the amount of *ESR1* mRNA (Fig. 4B). The ER protein level also reduced with estrogen and glyceollin I treatment (Supplementary Figs S1E and S2A), and the transcriptional activities of known ER target genes decreased with glyceollin I treatment (Supplementary Fig. S2B). Finally, glyceollin I inhibited LTED cell proliferation in time- and dose-dependent manners (Fig. 4C,D). Because LTED cells were deprived of estrogen, these inhibitory effects may be independent of estrogen.

We further characterized the effect of glyceollin I on three different cell states; LTED, MCF7, and IMR-90 (normal human fibroblast cell line derived from fetal lung) (Fig. 4E). Resveratrol inhibited all three cell types to a similar extent at the given condition. In contrast, glyceollin I inhibited LTED cells more effectively than IMR90 and MCF7 cells, suggesting its cell-type preference (Fig. 4E, also compare Fig. 4D and Supplementary Fig. S3A). Notably, both resveratrol and glyceollin I inhibited MCF7 cell growth at least mildly, unlike estrogen that promotes MCF7 growth^23^, despite the fact that all are structurally similar.

Resveratrol and glyceollin I also inhibited another endocrine resistant model, Tamoxifen resistant (TamR) cells derived from MCF7 cells (Supplementary Fig. S3B–E). Since the initial levels of the *Eleanors, ESR1* mRNA and ER protein were low in TamR cells, the direct effects of resveratrol and glyceollin I on TamR cells were not as evident as those on LTED cells (Supplementary Fig. S3B–D).

In conclusion, glyceollin I, isolated from soybeans activated with microorganisms, inhibited cell growth of LTED cell preferentially, suggesting a therapeutic potential against endocrine therapy-resistant breast cancer cells.

### Polyphenols induce apoptosis in LTED cells

Estrogen deprivation for a prolonged period primes MCF7 cells with apoptosis by estrogen^12,13,34^. The estrogen-induced apoptosis may be responsible for breast-tumor regression by therapy with high-dose estrogen. To investigate whether the apoptosis-inducing ability is conserved in resveratrol and glyceollin I, we performed FACS analysis of the LTED cells by Annexin V staining (Fig. 4F and Supplementary Fig. S4). Annexin V staining detects apoptotic cells in which phosphatidylserine is exposed to the outer plasma membrane. Administration of resveratrol or glyceollin I at 50 μM for 24 h increased apoptotic cells more than treatment with estradiol; a form of estrogen. This finding indicates that LTED cells are ready for apoptosis, which can be trigged with several types of polyphenols.

### Glyceollin I inhibits *Eleanors* independently of ER

**Figure 5:**
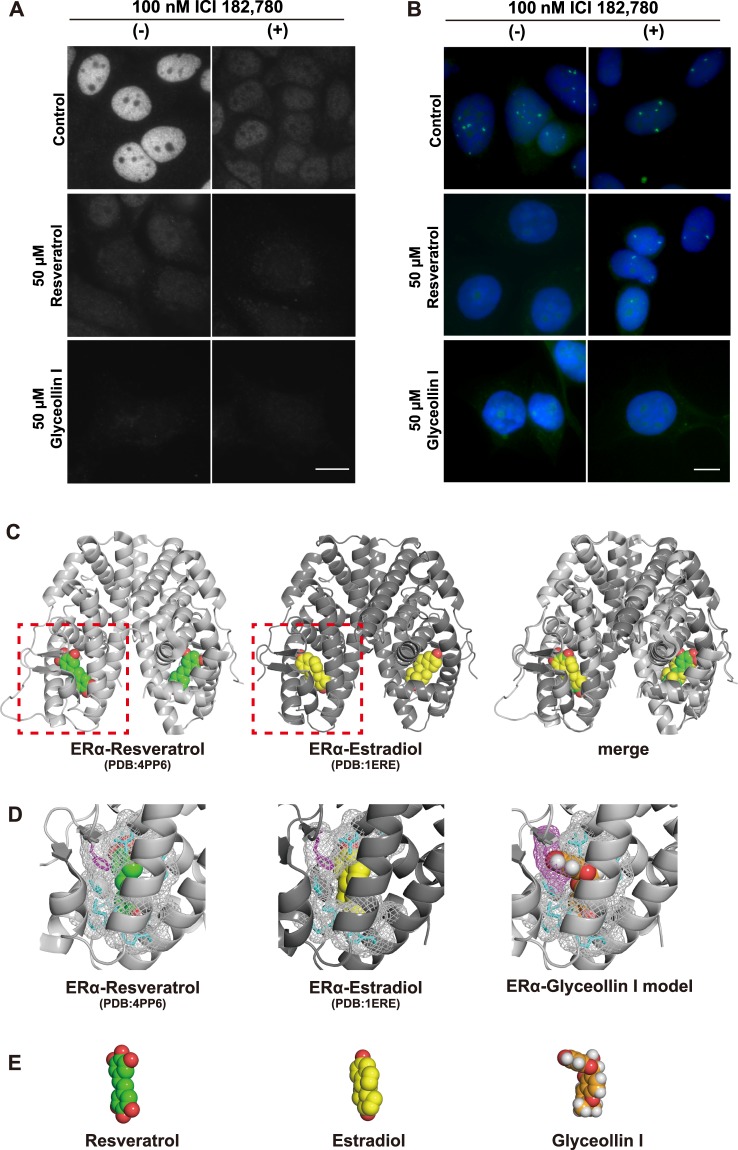
Glyceollin I exerts its inhibitory effect on *Eleanors* independently of ER. (**A**) Immunofluorescences confirmed degradation of ER in LTED nuclei by treatment with 100 nM ICI 182,780 for 72 h. Scale bar, 10 µm. (**B**) FISH analysis of *Eleanor* RNA cloud (green) in LTED nuclei (blue). *Eleanors were* inhibited by 50 μM resveratrol (left in the middle row), which was recovered by treatment with 100 nM ICI 182,780 (right in the middle row). On the other hand, the inhibition of *Eleanors* by 50 μM glyceollin I (left bottom) was maintained even under the identical ICI 182,780 treatment (right bottom). All of the drug treatments were for 72 h. Scale bar, 10 µm. (**C**) Comparison of the crystal structures of dimerized ligand binding domains of ERα bound by resveratrol (left)^22,35^ and estradiol (middle). The merged image is on the right. ERα is shown in a cartoon model. Resveratrol and estradiol are in a space-filling model. (**D**) Enlarged views of dotted areas in (**C**) are shown on the left and middle. The model structure of ERα bound to glyceollin I is shown on the right. The amino acids around the compounds are shown in a stick model, and their surfaces are shown in a mesh model. Phenylalanine (F404), shown in magenta, of ERα is the site where the steric hindrance with glyceollin I occurs (right). (**E**) Tertiary structures of resveratrol (left), estradiol (middle) and glyceollin I (right).

In estrogen-stimulated breast cancer cells, glyceollin I binds to ER and exerts anti-estrogenic effects^19,23,24^. To investigate how it inhibits *Eleanors* in the absence of estrogen, we treated LTED cells with 50 μM resveratrol or glyceollin I in combination with 100 nM ICI 182,780 which efficiently degrades ER proteins (Fig. 5A). Our FISH analysis showed that *Eleanor* RNA cloud in LTED cells was inhibited by 50 μM resveratrol (Fig. 5B, left in the middle row), which was rescued with ER degradation by 100 nM ICI 182,780 for 72 h (Fig. 5B, right in the middle row), similarly to our previous observation^10^. On the other hand, *Eleanor* inhibition by 50 μM glyceollin I (Fig. 5B, left bottom) was maintained under the same treatment (Fig. 5B, right bottom). Therefore, unlike estrogen and resveratrol, glyceollin I inhibited *Eleanors* independently of ER. Consistently, knockdown of ER with siRNA targeted to *ESR1* mRNA recovered the *Eleanor* RNA clouds that were regressed by resveratrol and estradiol, but not ones regressed by glyceollin I (Supplementary Fig. S5A,B).

Previous studies suggested that possible non-estrogen targets of glyceollin I include kinases and compounds of mTOR signaling that are important for the regulation of cell size and proliferation^26,28^. Our immunoblot also showed that the phosphorylation of Ribosomal Protein S6 Kinase, 70 kDa (p70S6), one of the best characterized downstream targets of mTORC1, was inhibited by glyceollin I in LTED cells (Supplementary Fig. S5C).

To understand how glyceollin I exerts its inhibitory effect differently from resveratrol and estradiol, we sought to visualize each compound bound to ERα. The structure of ERα bound with resveratrol was previously determined with the Y537S amino acid substitution^22^. This alteration stabilizes the structure, has little to no impact on ligand binding, and serves as a good tool for structural analysis^35^. The related mutation was detected in a population of LTED cells (Supplementary Fig. S5D), as previously reported^36^.

When we overlapped crystal structures of the ligand-binding domain of ERα complexed with resveratrol (PDB entry: 4PP6) and with estradiol (PDB entry: 1ERE), we found that both compounds positioned at the identical binding pocket (Fig. 5C). Then we modeled a tertiary structure of ERα bound to glyceollin I using the PyMOL program by replacing resveratrol in the ERα-resveratrol model (PDB entry: 4PP6) with glyceollin I (Fig. 5D). The results showed that the steric hindrance of glyceollin I occurs at phenylalanine (F404) of ERα. Comparison of the tertiary structures of resveratrol, estradiol, and glyceollin I (PubChem CID: 162807) also clarified the difference in glyceollin I, which is kinked and protrudes, whereas resveratrol and estradiol are plane (Fig. 5E). These findings suggest that glyceollin I binds ERα poorly or differently from estrogen and resveratrol, which may reflect the unique effect of glyceollin I.

## Discussion

In this study, we prepared a phytoalexin mixture from activated soybeans and identified glyceollin I to be a natural compound that preferentially inhibits the nuclear *Eleanor* RNA cloud, *ESR1* mRNA transcription, and cell proliferation in LTED cells. Similarly to estrogen, glyceollin I induced apoptosis of LTED cells, but with a different mechanism in which glyceollin I inhibited *Eleanors* independently of ER. Our study revealed the apoptosis-prone nature of LTED cells that appeared with glyceollin I treatment. This may represent the fragility of LTED and endocrine therapy-resistant breast cancer cells, which may be a therapeutic target.

Glyceollins are highly induced in soybean tissues with elicitors, as part of a self-defense system^19^. To date, seven kinds of glyceollins (glyceollin I~VII) have been identified, and they are biosynthesized from phenylalanine through daizen, isoflavon, glycinol, and glyceollins^31^. Although we found that glyceollin I (Fr. 6) was most effective in suppressing *Eleanors*, other fractions also showed moderate inhibitory effects (Fig. 2), representing candidate compounds for the modulation of events involving estrogen and ER. Therefore, legumes may be a rich source for health-promoting compounds. It should be noted, however, that glycinol, a precursor to glyceollin I is a potent phytoestrogen, which binds to ER to activate target gene transcription and promotes MCF7 cell viability^37^. Therefore, crude soybean extract is a mixture of ER agonist and antagonist, implying the importance of purification and precise characterization of each phytoalexin.

Glyceollin I has been reported to bind to ER, compete with endogenous estrogen, and suppress breast and ovarian tumorigenesis as an anti-estrogen^19,21,23,24,38^. The present and other studies showed that glyceollin I exerts additional antitumor activity in LTED (this study), triple-negative breast cancer^25,29^ and aromatase inhibitor (Letrozole)-resistant cell models^27^. Consistently, the binding affinity of glyceollin I to ER is significantly lower than one of estradiol^19,21,24,38^. For example, previously reported *in vitro* IC_50_ is 1.68 μM for glyceollin I, and it is 2.05 nM for estradiol, where the IC_50_ represents a concentration of unlabeled ligand required to replace 50% of the tracer from the ER *in vitro*^24^. Our structural comparison showed that estrogen and resveratrol bind to ER similarly, whereas steric hindrance occurs when resveratrol was virtually replaced with glyceollin I, suggesting that glyceollin I would bind to ER differently and function differently from estrogen and resveratrol (Fig. 5C–E).

It is interesting that glyceollin I preferentially suppressed proliferation of LTED cells, compared with MCF7 and IMR 90. The distinct property of glyceollin I suggests a novel cellular target in breast cancer under estrogen deprivation. One possibility is that glyceollin I inhibits the ER interaction with its partner proteins. The ER transcriptional activity is known to be modulated by its protein interactors, including FOXA1, GATA3 and GREB1^39^, and glyceollin I repressed the ER target genes (Supplementary Fig. S2B).

The efficacy of estrogen additive therapy on breast cancer in postmenopausal women has been demonstrated for a long time^40,41^ but with little appreciation, because the responses are paradoxical. Estrogen generally stimulates tumor cell growth, and therefore, removal of the ovaries in pre-menopausal women results in tumor regression. When the cells survive estrogen deprivation, several events occur, including upregulation of ERBB2, c-Myb, c-Myc, and MAP kinases and activation of the NOTCH, PI-3 kinase, mTOR growth, and FGER/REBB/AKT pathways^6,7,9,42,43^. In parallel, the cells are reprogrammed to be primed for estrogen-induced apoptosis, including upregulation of Fas and FasL, but they are maintained by anti-apoptotic activity^6,13^. Our present study revealed that LTED cells which adopted to the therapeutic environment, may be under a balance between cell proliferation and death, can be broken with glyceollin I, resveratrol, and estrogen in distinct manners. Our study provides insight into the acquisition of therapy resistance accompanied with an apoptosis-prone nature, or cancer fragility in breast cancer cells.

## Methods

### Cell culture and drug treatment

ER-positive human breast cancer MCF7 cells (ATCC) were cultured in RPMI 1640 (Sigma) supplemented with 10% fetal bovine serum (FBS, Corning). To establish LTED cells, MCF7 cells were grown in phenol red-free RPMI 1640 (Wako) containing 4% dextran-coated charcoal-stripped FBS (Thermo Fisher Scientific) for 2–4 months. To establish TamR cells, MCF7 cells were grown with 100 nM 4-OHT (hydroxy Tamoxifen, Sigma) for over 1 month. IMR 90 cells, human fibroblast cells derived from fetal lung, were maintained in DMEM/Ham’s F12 with l-glutamine and sodium pyruvate (Wako) supplemented with 10% FBS. The cells were treated with resveratrol (Sigma-Aldrich), Fr. 6, purified glyceollin I, ICI 182,780 (Tocris), or β-Estradiol (Wako) at indicated concentrations and time. As a control, the cells were treated with equal amounts of DMSO (nacalai tesque), a solvent for each drug.

### Preparation of phytoalexins from soybeans

Bulk soybeans were germinated in the dark, under 20% O_2_–0.025% CO_2_ with an intermittent supply of water at 27 °C every 3 for 48 h. They were then inoculated with *Aspergillus orzae* (25 g for 1 kg soybeans) for 4 days, followed by drying at 70 °C for 24 h, crushing, and extraction with 70% ethanol. The extracts obtained were analyzed by the Nexera X2 HPLC system (Shimadzu), which was equipped with a pump, autosampler, and PDA (photodiode array) detector. Sample (1 μL) was injected onto an Acquity UPLC BEH shield RP18 column (2.1 mm i.d. × 150 mm, 1.7 μm particle size; Waters) with an Acquity UPLC BEH shield RP18 VanGuard precolumn (2.1 mm i.d. × 5 mm, 1.7 μm particle size; Waters). Water acidified with 0.1% (v/v) acetic acid, eluent A, and acetonitrile acidified with 0.1% (v/v) acetic acid, eluent B, were used as solvents at a flow rate of 300 μL/min. The elution profiles for Fig. 1A,C were as follows: 0–2 min, linear gradient from 10% to 25% (v/v) B; 2–9 min, linear gradient from 25% to 50% (v/v) B; 9–12 min, isocratic on 50% B; 12–22 min, linear gradient from 50% to 100% (v/v) B; 22–25 min, isocratic on 100% B; 25–27 min, linear gradient from 100% to 10% (v/v) B; 27–29 min, isocratic on 10% (v/v) B. The temperatures of the autosampler and column oven were controlled at 15 °C and 35 °C, respectively. The PDA detector was set to monitor the 200–400 nm range.

### Large-scale preparation and purification of glyceollin I from soybeans

Purification of glyceollin I was performed as outlined in Fig. 1B. Soybean seeds (20 kg) were geminated and activated as described above and crushed into small pieces. Eighty liters of hexane was added to them, followed by vacuum filtration. The residuum was extracted with 80 L of 70% ethanol, vacuum filtered to concentrate, and applied to the open column (35 cm i.d. × 50 cm) with HP-20 synthetic adsorbents of Styrene body (Mitsubishi Chemical) to fractionate into Frs. A, B, and C, eluents with 120 L of water, 70% methanol, and 100% methanol, respectively. Materials in Fr. C were then applied to the open column (35 cm i.d. × 50 cm) with 20 kg Silica gel PSQ-100B (Fuji Silysia Chemical Chromatorex), and fractionated, in which fractions from Fr. 2 through 4 were eluted with 5 L of 70% hexane-30% ethyl acetate and Fr. 5 through 7 were eluted with 5 L of 50% hexane-50% ethyl acetate successively. Frs. 5 through 7, which contained glyceollins, were collected and applied to the open column (10 cm i.d. × 30 cm) with 1 kg Silica gel PSQ-100B and eluted with 2 L of 70% hexane-30% ethyl acetate, 4 L of 60% hexane-40% ethyl acetate, and 4 L of 50% hexane-50% ethyl acetate. The eluate was collected every 500 mL, and the 10th and 11th fractions were collected and applied twice onto a YMC-Pack ODS-A-HG HPLC column (50 mm i.d. × 250 mm, S-15 μm). The ODS HPLC system (Shimadzu) was equipped with a pump (SPD-10A, Shimadzu) and UV detector (SCL-10A, Shimadzu). As solvents, water acidified with 0.1% (v/v) TFA; eluent A, and acetonitrile acidified with 0.1% (v/v) TFA; eluent B were used for the first ODS column, and water acidified with 0.1% (v/v) TFA; eluent A, and methanol with 0.1% (v/v) TFA; eluent B were used for the second ODS column. Each time, the flow rate was 30–40 mL/min. The major peaks in Frs. 6 and 7 from the previous Silica gel PSQ-100 B column were separated at the first ODS column. Finally, the eluate was applied to the open columns (2.5 cm i.d. × 20 cm) with silica gel YMC-Pack ODS-A and a 50 g silica gel PSQ-100B column and eluted with 50% hexane-50% ethyl acetate. The resultant glyceollin I was 185 mg with more than 98% purity.

To analyze materials in fractions from Fr. 2 through 7 (Fig. 1C), the fractions were applied on an Acquity UPLC BEH shield RP18 column (2.1 mm i.d. × 150 mm, 1.7 μm particle size; Waters) with an Acquity UPLC BEH shield RP18 VanGuard precolumn (2.1 mm i.d. × 5 mm, 1.7 μm particle size; Waters). The elution profiles (Fig. 1C) were obtained with an Acquity UPLC BEH shield RP18 column (2.1 mm i.d. × 150 mm, 1.7 μm particle size; Waters), similarly to the isoflavonoid analysis described above and Fig. 1A.

### Nuclear magnetic resonance (NMR)

All measurements were carried out on an ECS-400 spectrometer (JEOL, Tokyo, Japan) operating at 400 MHz and 25 °C. Sample was dissolved in methanol-*d*_4_. ^1^H-NMR spectra were acquired by a single pulse sequence with water suppression using pre-saturation under the following conditions: X offset, 5.0 ppm; scans, 16; relaxation delay, 15 s; auto-gain and spinning. ^13^C-NMR spectra were acquired under the following conditions: X offset, 100 ppm; scans, 20000; relaxation delay, 2.0 s; auto-gain and spinning. All spectra were referenced to TMS (98.0 atom% D) at 0 ppm. Auto-shimming for each measurement was performed with field-gradient shimming at four scans at a receiver gain of 20. NMR data acquisition and analysis were performed using Delta software (version 1.1).

### Time-of-flight–mass spectrometry

Electrospray ionization (ESI)–time-of-flight–mass spectrometry (TOF-MS) analysis was carried out using micrOTOF II equipment (Bruker Daltonics, Bremen, Germany) in negative mode. The ESI conditions were as follows: drying gas, N_2_; flow rate, 8.0 L/min; drying gas temperature, 200 °C; nebulizing gas pressure, 1.6 bar; hexapole RF, 400 V; capillary voltage, −130 V; and mass range, 100–1000* m/z*. All data acquisition and analyses were controlled by Bruker Data Analysis 3.2 software. TOF-MS was externally calibrated using a sodium formate solution containing 10 mM sodium hydroxide in water-acetonitrile (1:1, v/v).

### Fluorescence *in situ* hybridization (FISH)

FISH was performed as previously described^10^. Briefly, LTED cells grown on coverslips were fixed with 4% paraformaldehyde and 0.5% Triton X-100 in PBS for 15 min at room temperature and then permeabilized with 0.5% saponin and 0.5% Triton X-100 in PBS for 20 min at room temperature. The coverslips were immersed in 20% glycerol in PBS for 30 min at room temperature and then subjected to four cycles of freezing and thawing. In a cycle, the coverslips were frozen in liquid nitrogen for 3 s followed by being thawed at room temperature. The cells were placed back in 20% glycerol and then treated with 0.1 N HCl for 15 min at room temperature. For denaturation and hybridization, the cells were incubated in hybridization mixtures (2 × SSC, 50% formamide, 10% dextran sulfate, 1 mg/mL tRNA, and 5–10 μg/mL probe DNA) at 37 °C for over 48 h with moisture in a Hybridizer (Dako). The BAC probes (RP11–450E24 for Figs 2, 4, 5, Supplementary Figs S1 and S3; RP1-63I5 for Supplementary Fig. S5) were labeled with digoxigenin (for Figs 2, 4 and 5) or biotin (for Supplementary Fig. S5) in a nick translation mixture (Roche) according to the manufacturer’s protocol. After hybridization, the coverslips were washed three times with 2 × SSC and 50% formamide at 37 °C for 5 min at room temperature, followed by another three washes with 2 × SSC at 37 °C for 5 min. The cells were further incubated with FITC-anti-digoxigenin (Roche, for Figs 2, 4, 5 and Supplementary Figs S1 and S3) or Alexa Fluor 488 Streptavidine (Invitrogen Molecular Probes, for Supplementary Fig. S5) to detect signals. DNA was counterstained with 5,6-diamidino-2-phenylindole (DAPI). Lastly, the coverslips were mounted on a slide with ProLong Gold Antifade (Thermo Fisher Scientific) for observation under a microscope.

### Immunofluorescence

Immunofluorescence was performed as previously described^10^. Briefly, LTED cells grown on coverslips were fixed with 4% paraformaldehyde in PBS for 15 min at room temperature and permeabilized with 0.2% Triton X-100 with 0.5% bovine serum albumin fraction V (BSA, Wako) in PBS for 5 min on ice. Then the cells were blocked with 0.5% BSA for 5 min for three times at room temperature and then incubated with anti-ER alpha antibodies (Santa Cruz Biotechnology, sc-543) overnight at 4 °C, and further incubated with secondary antibody Alexa Fluor 488-conjugated donkey anti-rabbit IgG (Molecular probes) for 60 min at room temperature. DNA was counterstained with 1 μg/mL DAPI, and the coverslips were mounted on slide glass as described for the FISH procedure.

### Microscopy

Images from FISH and immunofluorescence assays were captured with a microscope IX-71 (Olympus) that was equipped with a 60 × NA1.0 Plan Apo objective lens, a cooled charge-coupled device camera (Hamamatsu), and image acquisition software, Lumina Vision (Mitani). Images were taken under an identical condition for a set of drug treatments.

### Quantitative RT-PCR

Total RNA was isolated from cells with TRIzol (Thermo Fisher Scientific) and reverse transcribed into cDNAs with a Rever-Tra Ace PCR RT kit (TOYOBO). qPCR was performed with SYBR green fluorescence with the real-time PCR system, Step One Plus (Applied Biosystems). Values were normalized to glyceraldehyde-3-phosphate dehydrogenase (*GAPDH*) gene expression before calculating relative fold changes. Primer sequences are 5′-CAGGCCAAATTCAGATAATCG-3′ (forward) and 5′-TCCTTGGCAGATTCCATAGC-3′ (backward) to detect human *ESR1* mRNA, 5′-ATCAGCTGCTCGGACTTGCTG-3′ (forward) and 5′-TGAGCTCCGGTCCTGACAGATG-3′ (backward) to detect human *GREB1* mRNA, 5′-TGCTGTTTCGACGACACCGTT-3′ (forward) and 5′-AGGCAGATCCCTGCAGAAGT-3′ (backward) to detect human *TFF1* mRNA, 5′-AATGGAAGGGCAGCACAACT-3′ (forward) and 5′-CCAGCCTGACAGCACTTTCT-3′ (backward) to detect human *PGR* mRNA and 5′-ACACCCACTCCTCCACCTTT-3′ (forward) and 5′-TAGCCAAATTCGTTGTCATACC-3′ (backward) to detect human *GAPDH* mRNA.

### Immunoblotting

To prepare the total cell lysate, cells were dissolved in SDS sample buffer containing benzonase (Sigma). Proteins were separated by SDS– polyacrylamide gel electrophoresis and then transferred to a nitrocellulose membrane, Hybond-ECL (GE Healthcare). The membrane was blocked for 1 h with PBS containing 10% nonfat dry milk, and then incubated with primary antibodies against ERα (Santa Cruz Biotechnology, sc-543, 1:1,000), mTOR (Cell Signaling Technology, #2983, 1:1,000), Phospho-p70 S6 Kinase (Thr389) (Cell Signaling Technology, #9234, 1:1,000) and Actin (Sigma, A2103, 1:1,000) in Can Get Signal (nacalai tesque), overnight at 4 °C. The membrane was washed with PBS containing 0.3% Tween 20 three times for 5 min each, and incubated with horseradish peroxidase-conjugated secondary antibodies for 60 min. After the membrane was washed with PBS containing 0.3% Tween 20 three times for 5 min each, the signals were visualized with Chemi-Lumi One Ultra solution (nacalai tesque), and detected by Amersham Imager 600 (GE Healthcare).

### Cell growth assay

Relative cell proliferation was measured with colorimetric assays using the Cell Counting Kit-8 (Dojindo), according to the manufacture’s protocol. Briefly, cells with and without drug treatment were grown in 96-well plates, to which tetrazolium salt WST-8 was added. After a 1–4 h incubation at 37 °C in a humidified CO_2_ incubator, the degree of yellow colour of the media was measured with a microplate reader (BIO-RAD). Values of absorbance at 490 nm were normalized with absorbances at 630 nm. The values of DMSO-treated cells were set to 1. Biological triplicates were performed, and the values are reported as means ± SD. The *in vivo* IC_50_ values in Fig. 4D, and in Supplementary Figs S1 and S3 were calculated as the maximum drug concentrations to cause 50% inhibition of cell growth.

### Apoptosis assay

Cells were stained with Annexin-V-FLUOS Staining Kit (Roche) according to the manufacturer’s protocol. Briefly, the cells were detached from the dish by trypsin treatments and incubated in Annexin-V-FLUOS labeling solution for 15 min at room temperature and then applied to a FACS analyzer (BD FACSCanto™ II). For data analysis, the FlowJo (10.4.1 v) program was used.

### Comparison of protein structures of ERα bound for compounds

The protein structures of ERα bound to resveratrol (PDB entry: 4PP6) and estradiol (PDB entry: 1ERE) were created using PyMOL (http://www.pymol.org). 3D conformer data of (-)-glyceollin I (PubChem CID: 162807) were obtained in PubChem. The modeled structure of ERα bound to glyceollin I was created by replacing resveratrol in the ERα-resveratrol structure (PDB entry: 4PP6) with glyceollin I.

### Detection of the mutation in the *ESR1* gene

Genomic DNA was isolated from MCF7 and LTED cells, and the 817 bp DNA region containing the mutation hotspot in the ligand binding domain of ER^36^ was amplified by PCR, using TaKaRa LA Taq (TaKaRa). The amplified DNA fragment was labeled with BigDye Terminator V3.1 (ThermoFisher), and subjected to capillary sequencing. The results were displayed using 4Peaks. The primers used to amplify the DNA were as follows: forward primer 5′-CTTTCCCAGCTCCCATCCTAAAGTG-3′ and reverse primer 5′-TTATCTGAGCCCCAACCCATAGACT-3′.

### Statistical analysis

For cell growth and qPCR assays, experiments were performed at least three times. Values are means ± SD. Comparisons between groups were analyzed using the two-tailed Student’s *t*-test. A value of *p* < 0.05 was considered statistically significant.

## Electronic supplementary material

Supplementary materials
