# Supplementary material for: Endocrine therapy-resistant breast cancer model cells are inhibited by soybean glyceollin I through Eleanor non-coding RNA
Source: Sci Rep. 2018 Oct 12;8:15202. doi: 10.1038/s41598-018-33227-y (PMC6185934; doi:10.1038/s41598-018-33227-y)
Supplement: Supplementary file 1 — Supplementary materials [file 41598_2018_33227_MOESM1_ESM.pdf]

Supplementary materials

Endocrine therapy-resistant breast cancer model cells are inhibited by soybean glyceollin I through *Eleanor* non-coding RNA

Authors:

Tatsuro Yamamoto[1, 2, 6, #], Chiyomi Sakamoto[1, #], Hiroaki Tachiwana[2], Mitsuru Kumabe[1], Toshiro Matsui[3], Tadatoshi Yamashita[4], Masatoshi Shinagawa[5], Koji Ochiai[5], Noriko Saitoh[1,2]*, and Mitsuyoshi Nakao[1]*

Affiliations:

[1] Department of Medical Cell Biology, Institute of Molecular Embryology and Genetics, Kumamoto University, 2-2-1 Honjo, Chuo-ku, Kumamoto 860-0811, Japan
[2] Division of Cancer Biology, The Cancer Institute of JFCR, 3-8-31 Ariake, Koto-ku, Tokyo 135-8550, Japan
[3] Faculty of Agriculture, Graduate School of Kyushu University, 744 Mototoka, Nishi-ku, Fukuoka 819-0395, Japan
[4] Tokiwa Phytochemical Co. Ltd., 158 Kinoko, Sakura-shi, Chiba 285-0801, Japan
[5] Kajitsudo Co. ,Ltd. 1155-5, Tabaru, Mashiki-machi, Kamimashiki-gun, Kumamoto 861-2202, Japan
[6] Department of Oral and Maxillofacial Surgery, Faculty of Life Sciences, Kumamoto University, Kumamoto, Japan
[#] These authors equally contributed to this work.

Contact:

*Address correspondence to:
Noriko Saitoh, Ph.D. (noriko.saito@jfcr.or.jp)
Division of Cancer Biology,
The Cancer Institute of JFCR,
3-8-31 Ariake, Koto-ku, Tokyo 135-8550, Japan
Tel: +81-3-3570-0471

Mitsuyoshi Nakao, M.D., Ph.D. (mitnakao@kumamoto-u.ac.jp)
Department of Medical Cell Biology
Institute of Molecular Embryology and Genetics, Kumamoto University
2-2-1 Honjo, Chuo-ku, Kumamoto 860-0811, Japan
Tel: +81-96-373-6800

Supplementary Table 1

Ct values of Figure 2B

		Control	Resveratrol	Fr.4	Fr.7		Control	Fr.2	Fr.3		Control	Fr.6
ESR1	Ave. (n=3)	20.33	26.10	24.77	25.47		19.40	20.13	22.91		20.24	25.24
	SD	0.95	0.48	0.40	0.81		0.77	0.62	1.50		0.53	0.43
GAPDH	Ave. (n=3)	15.61	17.03	16.49	17.65		15.67	15.94	16.50		15.52	16.96
	SD	0.24	0.17	0.35	0.95		0.38	0.12	0.70		0.51	0.33

Supplementary Table 2

Ct value of Figure 4B

		Control	Resveratrol	Fr.6	Glyceollin I
ESR1	Ave. (n=3)	17.95	21.41	21.43	21.19
	SD	0.41	0.34	0.70	1.37
GAPDH	Ave. (n=3)	15.96	16.15	16.07	15.95
	SD	0.14	0.50	0.99	0.50

Supplementary Figure S1. Estradiol inhibited *Eleanors* and LTED cell proliferation.

(A) FISH analysis showing that the *Eleanor* RNA cloud regressed in LTED cells treated with high concentrations of estradiol. Scale bar, 10 µm. **(B)** The *ESR1* mRNA level was decreased by estradiol in LTED cells. The *ESR1* mRNA level was measured by qRT-PCR. Values were normalized against *GAPDH* mRNA, and values for cells treated with control (DMSO) in LTED cells were set to 1. The bars represent the means ± S.D. $n = 3$, $^{**}p < 0.01$. **(C)** LTED cell proliferation was inhibited by high concentrations of estradiol. The values represent the means ± S.D. $n = 3$, $^{**}p < 0.01$. **(D)** LTED cell growth was inhibited by estradiol in a dose-dependent manner. The values represent the means ± S.D. $n = 3$. **(E)** Immunoblot showing that the ER protein levels were decreased in LTED cells by estradiol treatment. Actin was used as an internal control. MW, molecular weight. Full-length blots are presented in Supplementary **Fig. S6**.

Supplementary Figure S2. Glyceollin I decreased the ER protein level and its transcriptional activity in LTED cells.

(A) Immunoblot showing that the ER level was reduced by glyceollin

I in LTED cells. Actin was used as an internal control. Full-length blots are presented in Supplementary **Fig. S6**. **(B)** Quantitative RT-PCR showing that the expression levels of the ER target genes (*GREB1*, *TFF1*, *PGR*) decreased with glyceollin I treatment. The values for the control treatment were set to 1. The bars represent the means ± S.D. $n = 3$, *$p < 0.05$, **$p < 0.01$.

Supplementary Figure S3. The effects of resveratrol and glyceollin I on MCF7 and tamoxifen-resistant cells.

(A) MCF7 cells were inhibited with resveratrol and glyceollin I in a dose-dependent manner. The effect of glyceollin I in MCF7 cells was lower than in LTED cells, while the effect of resveratrol was comparable in MCF7 and LTED cells (see **Fig. 4E**). **(B)** RNA FISH showing that the *Eleanor* RNA cloud was not detected in TamR cells. Scale bar, 10 µm. **(C)** The level of *ESR1* mRNA decreased slightly by the glyceollin I treatment of TamR cells. Quantitative RT-PCR was performed to measure the relative *ESR1* mRNA levels in the indicated cells. Values were normalized against *GAPDH* mRNA, and values for cells treated with DMSO (control) in TamR cells were set to 1. The bars represent the means ± S.D. $n = 3$, *$p < 0.05$; **$p < 0.01$. **(D)** Immunoblot showing that the ER protein levels slightly decreased with the glyceollin I

treatment of TamR cells. Actin was used as a loading control. Full-length blots are presented in Supplementary **Fig. S6**. **(E)** TamR cell proliferation was inhibited by glyceollin I. The cell growth was measured with a colorimetric assay at 490 nm (Kit-8). The values represent the means ± S.D. $n = 3$, ***$p < 0.001$.

Supplementary Figure S4. Resveratrol, estradiol and glyceollin I induced apoptosis in LTED cells.

FACS analysis of Annexin V positive cells. Treatment of LTED cells with resveratrol, estradiol and glyceollin I increased a population with intense labeling with Annexin V, as indicated by bars. Data from triplicate experiments are shown.

Supplementary Figure S5. Glyceollin I exerts its inhibitory effect on *Eleanors* independently of ER.

(A) Immunofluorescences confirmed ER degradation by RNAi knockdown. LTED cells were treated with siRNA targeted to the *ESR1* mRNA (si*ESR1*) or control siRNA (si*GL3*) for 24 h, and ER was stained with antibodies. Scale bar, 10 µm. **(B)** FISH analysis of the *Eleanor* RNA cloud (green) in LTED nuclei (blue). *Eleanors* were inhibited by 50 µM resveratrol, estradiol, or glyceollin I in the control knockdown (left,

from the second to the bottom row). *Eleanors* were recovered by ER knockdown in resveratrol or estradiol-treated LTED cells (right, the second or third row). On the other hand, the inhibition of *Eleanors* by glyceollin I was maintained even in the absence of ER (right, bottom row). All drug treatments were for 24 h. Scale bar, 10 μm. **(C)** The activity of the mTOR pathway components was decreased by resveratrol and glyceollin I treatments in LTED cells. Immunoblotting revealed that the phosphorylation level of the mTOR substrate (phospho-p70 S6 kinases) decreased with glyceollin I treatment (middle panel), while the mTOR protein level did not change (top panel). Actin was used as a loading control (bottom panel). Full-length blots are presented in Supplementary Fig. S6. **(D)** The A to G mutation corresponding to ESR1^{Y537C} was detected in a sub-population of LTED cells (black arrow). DNA sequencing profiles obtained from Sanger capillary electrophoreses are shown.

Supplementary Figure S6. Original immunoblot images.

Uncropped immunoblots for Figs S1E, S2A, S3D and S5C are shown. Separate blots are delineated with black lines.

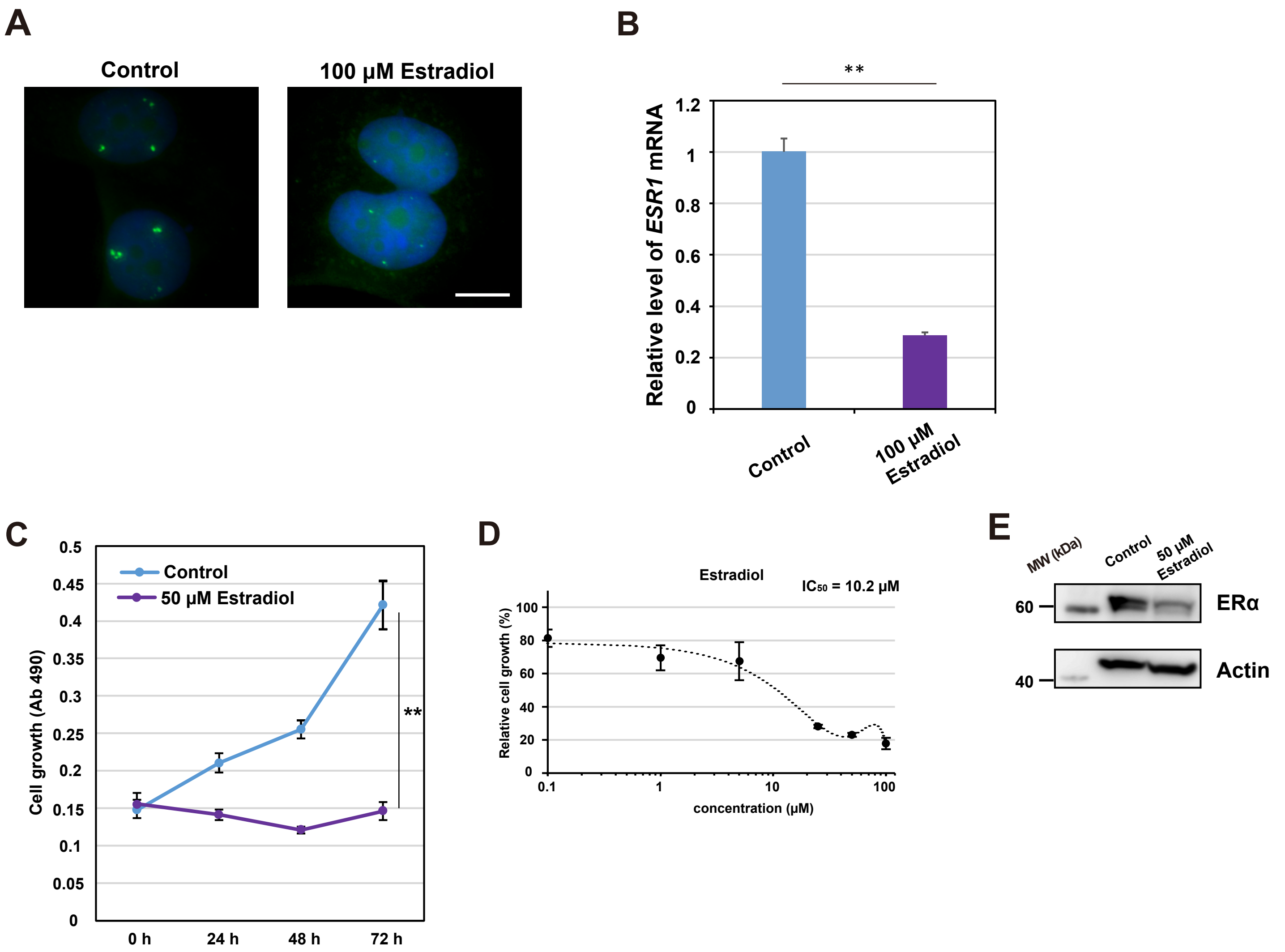

Supplementary Figure S1. Yamamoto et al

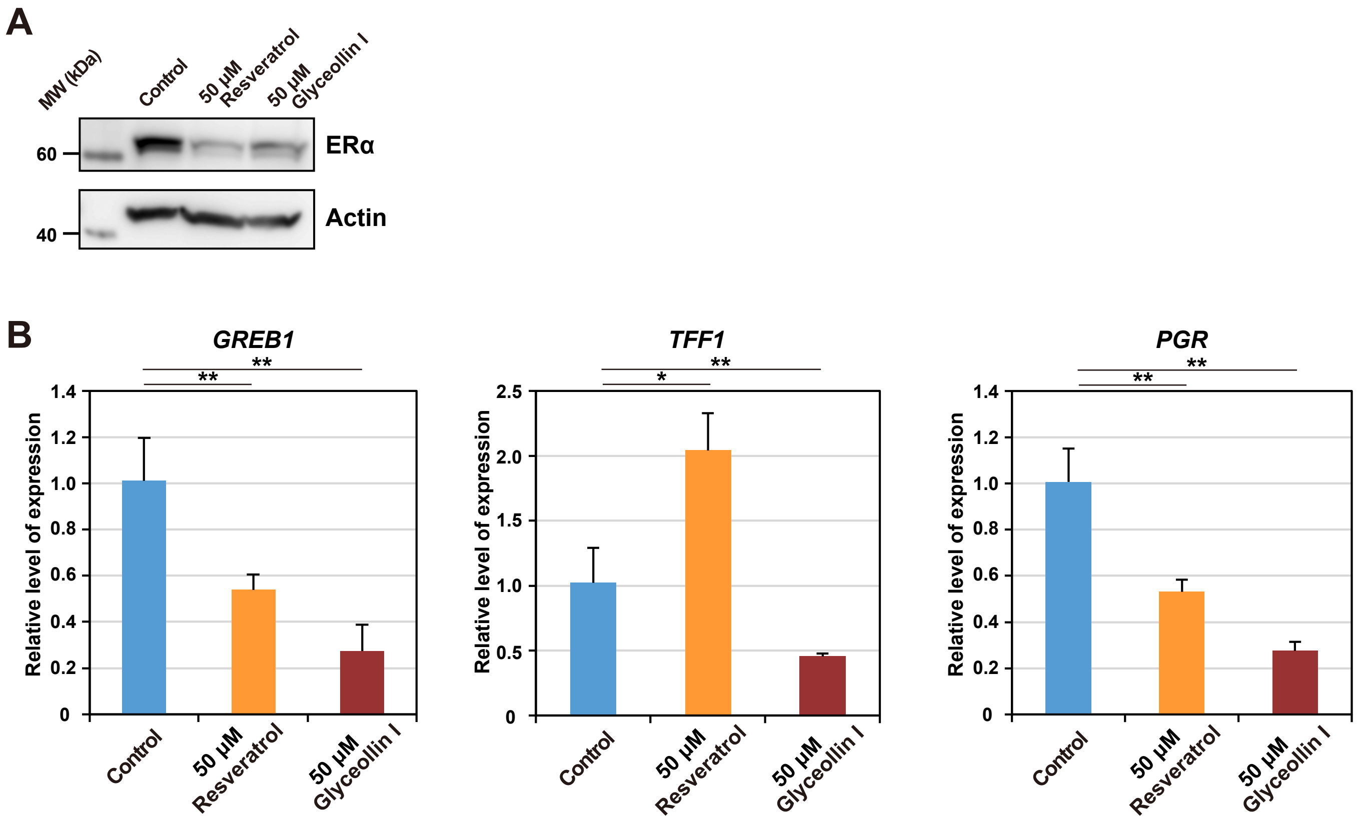

Supplementary Figure S2. Yamamoto et al

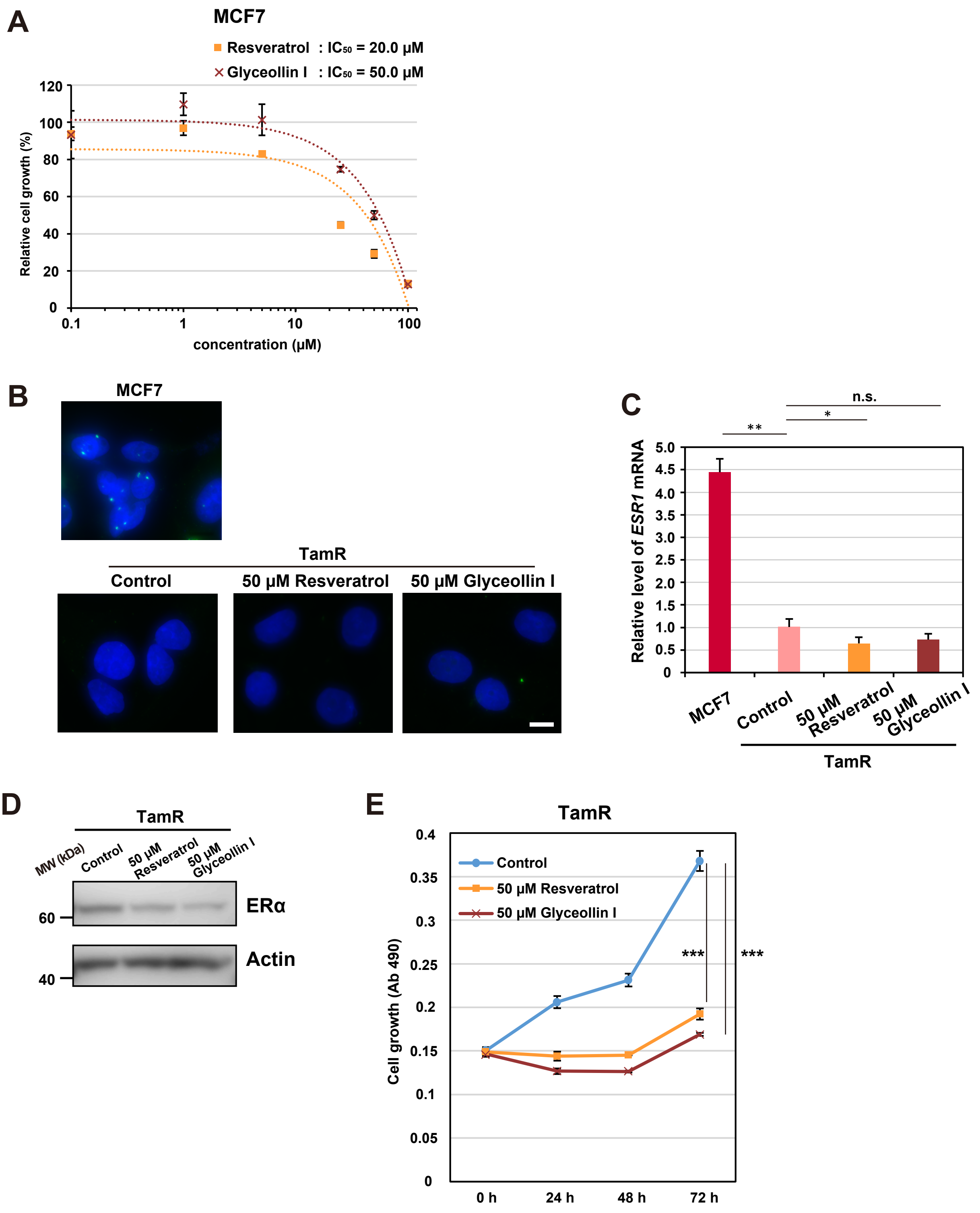

Supplementary Figure S3. Yamamoto et al

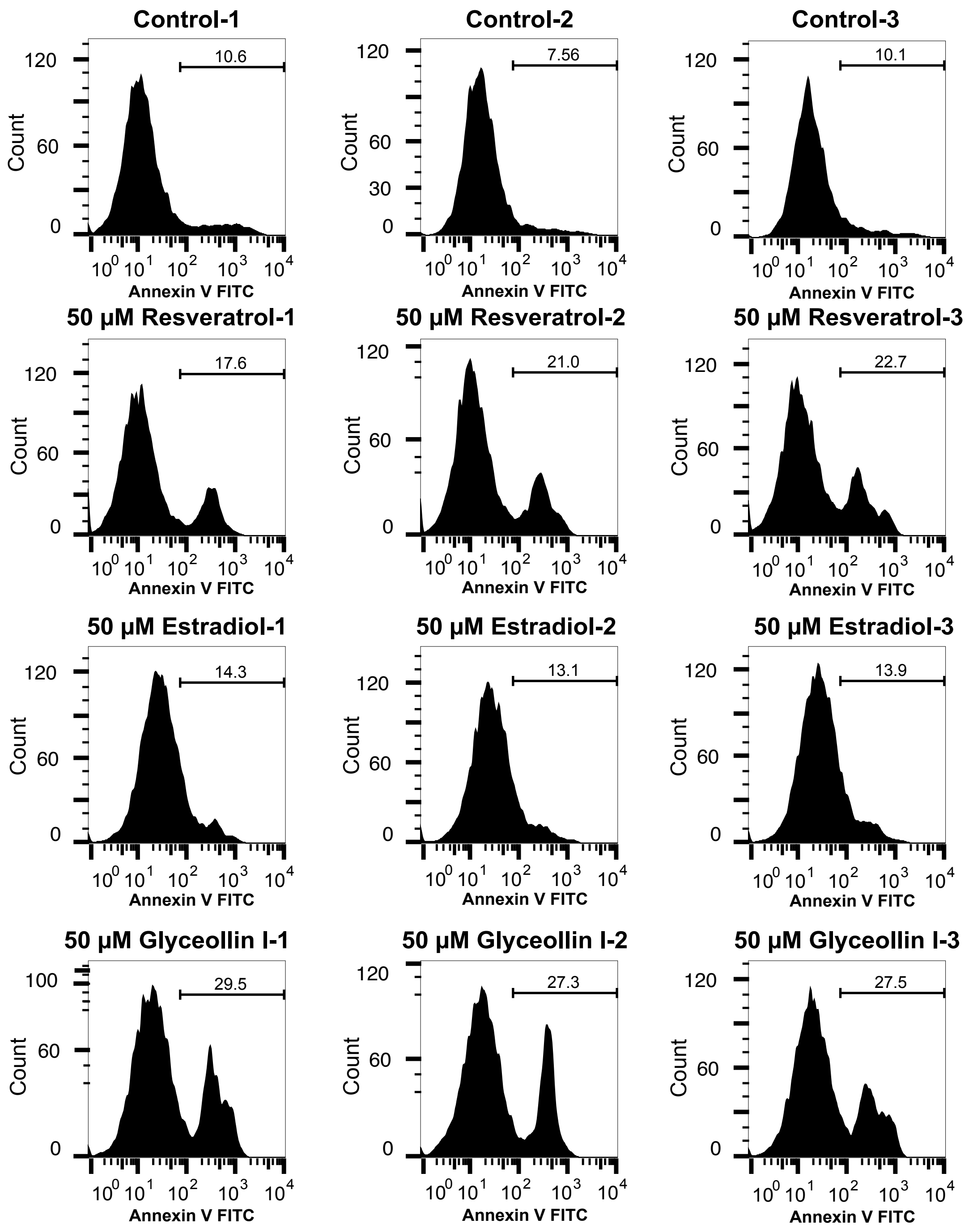

Supplementary Figure S4. Yamamoto et al

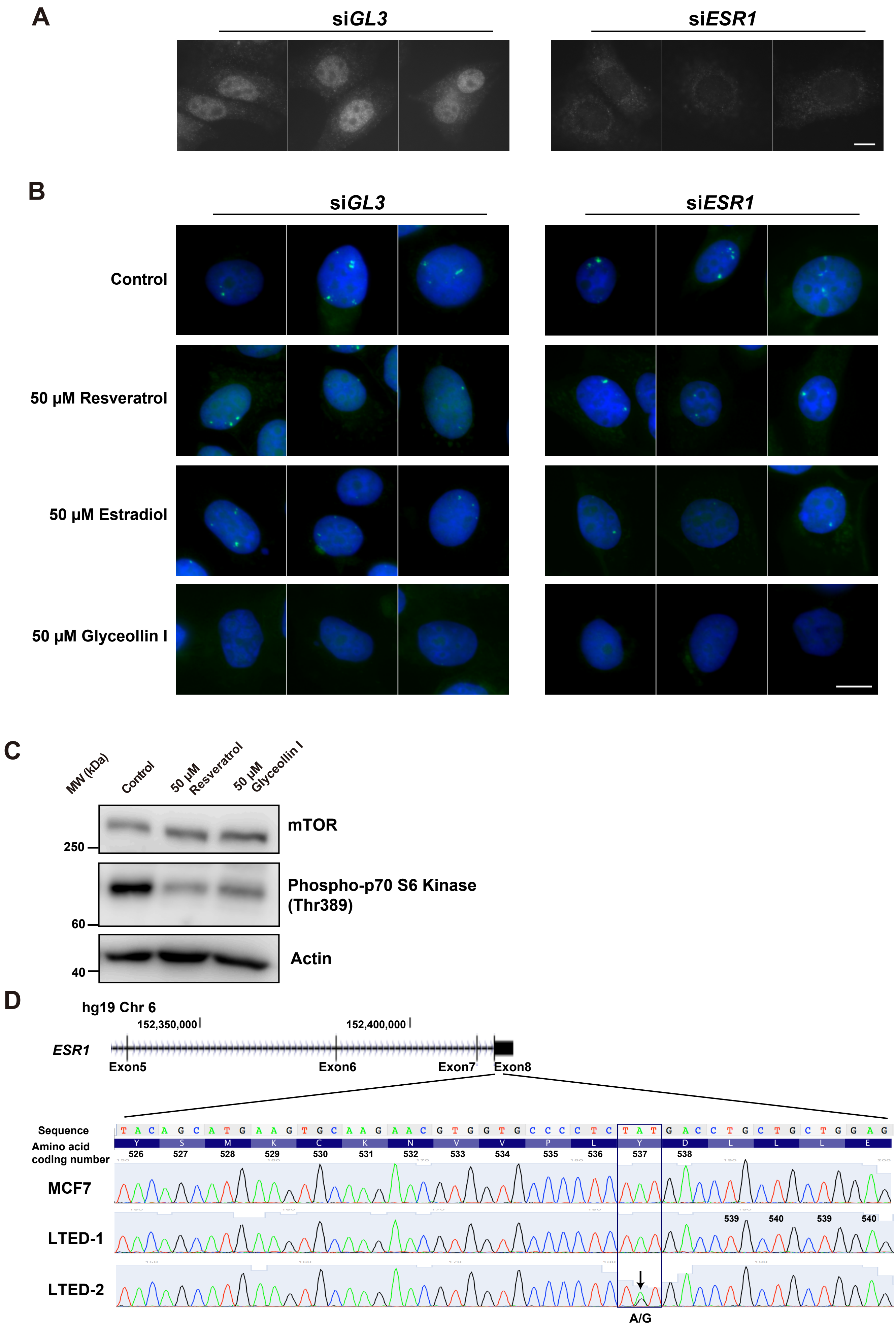

Supplementary Figure S5. Yamamoto et al

Supplementary Figure S1E, S2A

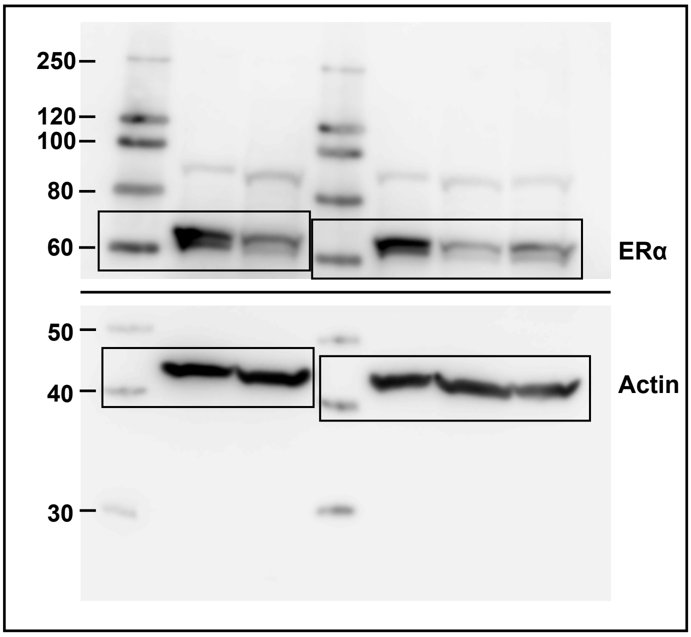

Supplementary Figure S3D

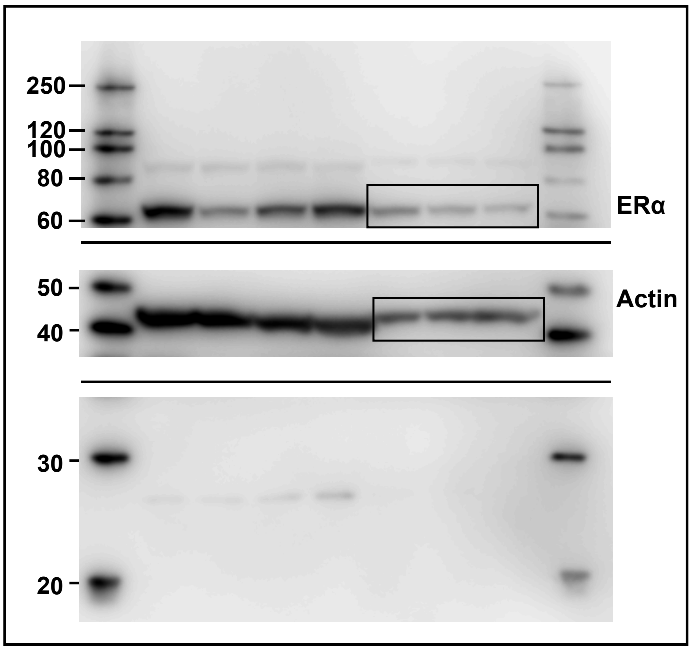

Supplementary Figure S5C

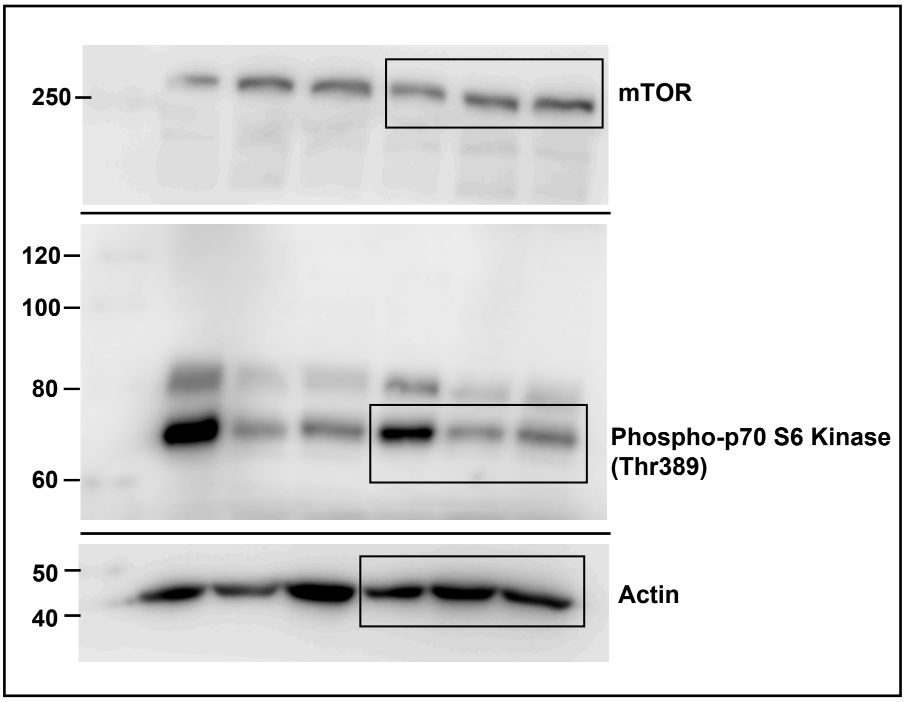

Supplementary Figure S6. Yamamoto et al
